# HIV-1 Subtype D Infections among Caucasians from Northwestern Poland—Phylogenetic and Clinical Analysis

**DOI:** 10.1371/journal.pone.0031674

**Published:** 2012-02-16

**Authors:** Miłosz Parczewski, Magdalena Leszczyszyn-Pynka, Dorota Bander, Anna Urbanska, Anna Boroń-Kaczmarska

**Affiliations:** Department of Infectious Diseases and Hepatology, Pomeranian Medical University, Szczecin, Poland; McGill University AIDS Centre, Canada

## Abstract

**Background:**

HIV-1 subtype D infections, which are associated with a faster rate of progression and lymphocyte CD4 decline, cognitive deficit and higher mortality, have rarely been found in native Europeans. In Northwestern Poland, however, infections with this subtype had been identified. This study aimed to analyze the sequence and clinical data for patients with subtype D using molecular phylogeography and identify transmission clusters and ancestry, as well as drug resistance, baseline HIV tropism and antiretroviral treatment efficacy.

**Methods:**

Phylogenetic analyses of local HIV-1 subtype D sequences were performed, with time to the most recent common ancestor inferred using Bayesian modeling. Sequence and drug resistance data were linked with the clinical and epidemiological information.

**Results:**

Subtype D was found in 24 non-immigrant Caucasian, heterosexually infected patients (75% of females, median age at diagnosis of 49.5 years; IQR: 29–56 years). Partial *pol* sequences clustered monophyletically with the clades of Ugandan origin and no evidence of transmission from other European countries was found. Time to the most common recent ancestor was 1989.24 (95% HPD: 1968.83–1994.46). Baseline drug resistance to nucleoside reverse transcriptase inhibitors was observed in 54.5% of cases (mutations: M41L, K103N, T215S/D) with evidence of clustering, no baseline integrase or protease resistance and infrequent non-R5 tropism (13.6%). Virologic failure was observed in 60% of cases and was associated with poor adherence (p<0.001) and subsequent development of drug resistance (p = 0.008, OR: 20 (95%CI: 1.7–290).

**Conclusions:**

Local subtype D represented an independently transmitted network with probably single index case, high frequency of primary drug resistance and evidence of transmission clusters.

## Introduction

Phylogenetic analyses have been widely implemented in molecular epidemiology of HIV infection and allowed not only to understand genetic sequence variability, but also to trace the flow of the variants and drug resistant clades as well as to infer a rate of molecular evolution to date the ancestry [1]. Clusters of sexual transmissions have been reconstructed and dated using sequence data obtained for drug resistance genotyping, providing information on the transmission patterns and allowing to identify distinct epidemiological entities [2]. Tracking changes in HIV epidemics related to subtype diversity and drug resistance is important in Europe, where genetic diversity of HIV is rising, with a trend to increase the prevalence of non-subtype B infections [3]. This variation is related either to immigration or the founder effect with subsequent rapid spread within the populations at risk. Migration from Africa and South America has been identified as a major source of diversity in Western Europe [4] with multiple, continuous inflow of the new clades into the population and common association with heterosexual transmissions [5], [6], [7]. Onward infections with the non-subtype B clades reported in Europe were mostly acquired from the partners originating from countries of the higher HIV prevalence [8], [9], however unique epidemics among drug users have also been noted [10]. Such explosive outbreaks in countries where drug injection is, or used to be common, were observed both for subtype B and non-B clades. This often results in high prevalence of a variant uncommon elsewhere, as was the case in Estonia and Kaliningrad region of Russia [11], [12]. Studies have also revealed a nosocomial epidemics with subtype F, of the Angolan ancestry, among children in Romania due to a distribution of contaminated needles and blood products [13], [14].

Acquisition of non-B subtypes may be associated with high prevalence of minor drug resistance mutations in untreated individuals and changes in virus tropism [15], [16], [17], [18]. Prevalence of major protease and reverse transcriptase mutations is similar across the variants, however both virologic and immunologic treatment efficacy is similar to the subtype B [19], [20]. In general, frequency of primary drug resistance differs locally in Europe, often being associated with transmission clusters, fluctuates over time and is usually limited to a single class of antiretrovirals [21], [22], [23], [24]. In Poland the prevalence of transmitted drug resistance in the years 2006–2009 ranged from 3.9% to 7.4% [25]. The predominant subtype B was observed in 95.8% of the newly diagnosed HIV infections [25].Of note, sexual transmissions now tend to be the most common in Poland, which is a significant change of epidemiological pattern from the earlier dominant injection drug use related transmissions [26]. We have previously identified higher prevalence of non-B subtypes, with a number of subtype D clades in the local population from Northwestern Poland (21%). These variants have been associated with more advanced HIV disease and more common heterosexual transmission route if compared to the subtype B [27].

In this study the sequences and clinical data from a cluster of locally diagnosed individuals infected with subtype D were analyzed. Firstly, we used molecular phylogeography to trace the import of subtype D into Poland, infer between-host phylogenies, indentify transmission clusters and determine ancestry with molecular clock methods. Secondly, baseline drug susceptibility (protease, reverse transcriptase and integrase) and V3 based predicted viral tropism were assessed. Finally, treatment efficacy among patients infected with this variant and secondary drug resistance were summarized.

## Results

### Group characteristics

Of the total of 190 patient sequences sampled so far in at the Department of Infectious Diseases and Hepatology, Szczecin, Poland HIV-1 subtype D was found in 24 (12.63%) cases. The first case of the infection with this variant was confirmed in a year 1994 in a young female, who had never travelled outside Poland. All cases were of Caucasian origin with the predominance of females (n = 18, 75%) and a median age at diagnosis of 49.5 years (IQR: 29–56 years). For only one patient presumed infection from a partner travelling overseas (sailor) was recorded, however the source sample was not available. Travel history for this contact is not specific, with frequent worldwide travels and no clear African connection. In the remaining cases no history of travel outside Europe was noted.

For all cases heterosexual transmissions were recorded, and patients were HCV antibody negative at HIV diagnosis. In one case HCV infection with antibody production was observed in the third year of observation. Patients tended to be diagnosed at symptomatic stages of the HIV infection (n = 21, 87.5%) with a low baseline and nadir lymphocyte CD4 counts (median 62 cells/µl [IQR: 23–246 cells/µl] and 35 cells/µl [IQR: 10–144 cells/µl, respectively) and a high baseline HIV-1 viraemia (median of 5.6 log HIV-1 copies/ml [IQR: 4.9–6 log HIV-1 copies/ml]).

Total observation period in the group was 116 person-years with a mean observation time of 57.82 months (95% CI: 35.37–80.27). In fourteen cases (58.3%) AIDS was diagnosed within the period of observation. Mortality rate was 3.44 per 100 person-years. Of the four deaths recorded, three were AIDS-related and occurred within the first year of observation; two cases of large B-cell lymphoma, one *Pneumocystis jirovecii* pneumonia and the fourth related to the cardiac event eight years after HIV diagnosis.

### Phylogeography and dated phylogeny of local subtype D

For the phylogeographic and dated phylogeny the earliest possible sample from every patient was selected and sequenced. The majority of samples were obtained from treatment-naive patients (n = 22, 91.7%), the remaining two samples were from patients on ARV.

**Figure 1:**
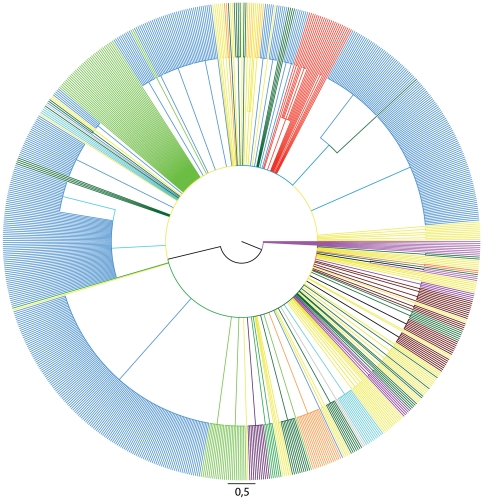
Relationships between the subtype D sequences inferred using maximum likelihood phylogeny (GenBank deposited *pol* sequences). Country-specific sequences are marked with the same color: red – Poland, blue – Uganda, green – Tanzania, yellow – Europe (except Poland), brown – Cameroon, magenta - Senegal, cyan- Sudan, dark green – other African countries, violet – South America, grey – Asia, orange – North America.

In the maximum likelihood tree all sequences from Poland formed a monophyletic cluster nested within the Ugandan sequences (figure 1, shown in red). Of note, subtype D sequences found in various European countries (figure 1, marked in yellow) were interspersed among various lineages of African origin, with no clustering with Polish sequences. Branch lengths of our sequences indicated independent evolution of the imported variant without admixture of African variants or transmission to other European countries.

**Figure 2:**
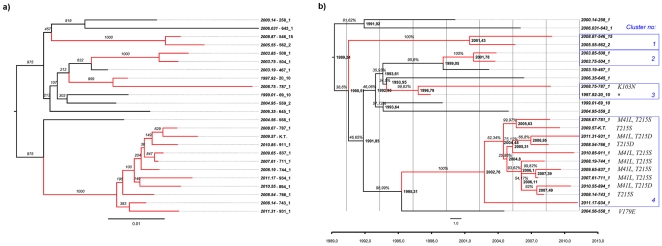
Phylogenetic trees of the subtype D sequences from Northwestern Poland. Figure a - maximum likelihood tree with bootstrap values for 1000 replicates drawn at the branches. Figure b – time scaled Bayesian MCMC tree. On the tree branches estimated time to the most recent common ancestor (tMRCA) and posterior probabilities expressed as percentage are shown. For both figures clustered sequences are marked in red and four identified clusters indicated as blue boxes and numbered are drawn on the right. Drug resistance mutations are marked at the tip nodes after the sequence identifier. *source patient for the transmission of the drug resistance within the cluster.

**Figure 3:**
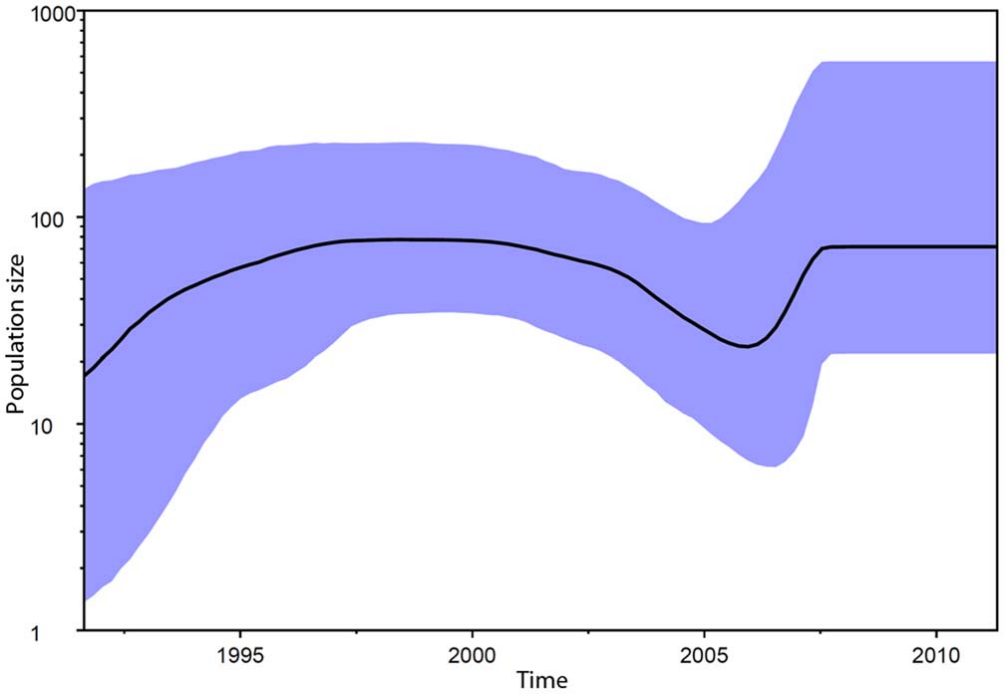
Bayesian skyline plot for estimation of the number of subtype D HIV-1 cases in the local population. 95% CI are marked in blue. Y-axis: predicted number of cases (log scale), X-axis: timescale (years).

In the reconstruction of the subtype D phylogeny, the maximum likelihood tree (figure 2a) and the Bayesian clade credibility tree (figure 2b) with dated ancestry had a similar topology. Time to the most recent common ancestor inferred using the relaxed clock model with GTR+γ+Γ was in accordance to the transmission dates from the patients' documentation. Four transmission sub-clusters may be observed with both maximum likelihood bootstrap values and Bayesian posterior probability >90% (figure 2b, sub-clusters numbered 1–4, blue boxes). Using Bayesian Monte Carlo Markov Chain, dating to the time to the most recent common ancestor (tMRCA) for all regional sequences was the year 1989 (table 1). By the year 1994, which was the date of the first confirmed infection with the subtype D, the virus was probably already circulating locally. Bayesian skyline plot (figure 3) indicated that a population size has been fairly stable over time with a plateau in the last three years.

**Table 1:** Time to the most recent common ancestors (tMRCA) for the entire cluster and sub-clustered sequences.

	tMRCA (years)	95% HPD (value)	95% HPD (years, calibrated to the youngest tip)
All sequences	1989.24	16.85–42.48	1994.46–1968.83
cluster 1	2001.43	6.38–11.98	2004.93–1999.33
cluster 2	1999.05	10.38–19.16	2000.93–1992.15
cluster 3	1996.79	13.39–17.15	1997.92–1994.16
cluster 4	2002.76	5.95–12.79	2005.36–1998.52

### Clusters of drug resistance

Four observed clustered sequences were linked epidemiologically, which was reflected in the patients' records – cases within the sub-clusters 1–3 were sexual partners. Baseline drug resistance was observed in 12 of the 22 antiretroviral naive patients (54.5%) with pretreatment genotyping available. Primary drug resistance was the most common within the fourth, the youngest (tMRCA in the 2002 [95% HPD: 2005.36–1998.52]) sub-cluster (10/11 cases, 90.9%), with nucleoside reverse transcriptase (NRTI) mutations observed. Ten patients within this sub-cluster were female and one male; epidemiological data from patients' records suggested possible sexual contacts between the man and at least three women. Median age in this subgroup was 56 years (IQR: 53–60 years); all patients were diagnosed with clinical features of immunodeficiency (category B and C according to the CDC 1993).

**Figure 4:**
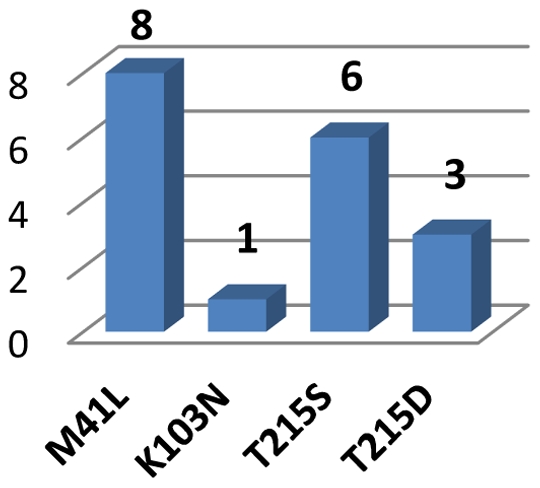
Frequency of baseline drug resistance mutations among treatment-naive patients.

In two patients non-nucleoside reverse transcriptase (NNRTI) drug resistant virus was observed in pretreatment samples (as shown in figure 2b). It was possible to identify the source of infection for one of these cases (sub-cluster 3, source marked with asterisk, data from the sequencing of the sample taken after virological failure, sequence not included in the tree). Frequency of the baseline drug resistance has been shown in figure 4. No baseline integrase or protease resistance was observed in the entire group. Non-R5 tropism was assigned in three (13.6%) baseline, treatment-naive samples with geno2pheno false positive rate (FPR) threshold set to 10% (one if FPR of 5.75% was used), sequences with FPR<10% were not clustered.

### Response to antiretroviral treatment and secondary drug resistance

**Figure 5:**
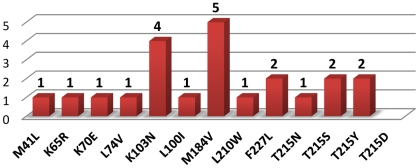
Frequency of secondary drug resistance mutations in patients failing antiretroviral therapy.

The group of 20 (83.3%) patients were commenced on stable antiretroviral therapy. Of the remaining one patient refused consistently, one was lost to follow-up within a month following the HIV diagnosis, one died before the treatment was introduced and one died within the first month of treatment. The median for the highest recorded lymphocyte CD4 count on cART was 369.5 (IQR: 294.25–596.5) cells/µl, with only five (25%) patients maintaining stable CD4 count >500 cells/µl for at least six months during the follow-up period. In 10 (50%) cases clinician recorded poor adherence, while pharmacy records confirmed <90% adherence in 9 (45%) patients. In 12 (60%) cases the primary therapy was virologically successful (table 2), and as expected, failure was associated with poor or <90% adherence (p<0.001) and development of drug resistance (p = 0.008, OR: 20 (95%CI: 1.7–290). Secondary drug resistance was noted in all eight failing patients with NRTI (M184V, T215 complex) and NNRTI (K103N) being the most common mutations (figure 5). No secondary protease drug resistance mutations were found in the group. For virologically failing patients non-R5 tropism was assigned in two of these cases (25%, geno2pheno algorithm with FPR 10%); if FPR 5.75% was used the non-R5 tropism would be called in one (12.5%) patient.

**Table 2:** Drug resistance and treatment efficacy in the group infected with subtype D.

No	Patient ID	Gender (F- female, M-male)	Year HIV diagnosed	Transmitted drug resistance (RT codons)	Virologic success of the first therapy	Adherence (clinician assigned)*	Secondary drug resistance (accumulated, RT codon position)	Virological control achieved on
**1**	20	F	1994	none	no	46% (poor)	L74V, K103N, L100I	Never achieved
**2**	69	F	1996	Not available	No	50% (poor)	M184V, T215N, T215S, T215Y, K103N	NRTI+PI+InI
**3**	258	F	1997	none	Treatment naive			
**4**	467	F	2003	none	yes	100% (adequate)	None	NRTI+PI
**5**	504	M	2003	none	no	45,69% (poor)	K103N	NRTI+PI
**6**	508	F	2003	none	yes	86,89% (medium)	None	NRTI+NNRTI
**7**	546	M	2004	Not available	no	78,46% (poor)	M184V	NRTI+PI
**8**	558	M	2004	V179E	Death before assessment			
**9**	559	F	2004	none	no	19,98% (poor)	M184V	Never achieved
**10**	562	F	2004	none	yes	62,11% (poor)	None	NRTI+NNRTI
**11**	643	M	2006	none	no	81,76% (poor)	K65R, K70E, M184V, V106A, F227L	NRTI+NNRTI, PIs+NNRTI, NRTI+PI
**12**	645	F	2006	none	no	89,49 (poor)	K103N, P225H, F227L, M184I	NRTI+NNRTI, NRTI+FI+PI
**13**	711	F	2007	M41L, T215S	yes	99,35% (adequate)	None	NRTI+PI
**14**	743	F	2008	M41L	yes	97,44% (adequate)	None	NRTI+NNRTI, NRTI+PI, NRTI+InI
**15**	744	F	2008	M41L, T215S	yes	97,33% (adequate)	None	NRTI+PI
**16**	766	F	2008	T215D	yes	96,45% (adequate)	None	NRTI+NNRTI, NRTI+InI
**17**	781	M	2008	M41L, T215S	yes	93,53% (adequate)	None	NRTI+NNRTI
**18**	787	M	2008	K103N	Treatment naive, lost to follow-up			
**19**	837	F	2009	M41L, T215S	yes	100,00% (adequate)	None	NRTI+PI
**20**	894	F	2010	M41L, T215D	yes	94,49% (adequate)	None	NRTI+PI, NRTI+InI
**21**	911	F	2010	M41L, T215S	yes	98,55% (adequate)	None	NRTI+InI
**22**	931	F	2011	M41L, T215D	Yes	100,00% (adequate)	None	NRTI+InI
**23**	934	F	2011	none	no	94,29% (poor)	M184I, Y181C	NRTI+PI
**24**	KT	F	2009	T215S	Treatment naive, death before assessment			

*Percentage expressed adherence based on the number of months of medications dispensed by the number of months of follow-up; clinician assigned adherence based on the patient's statement regarding missed doses and treatment interruptions.

Interestingly, the treated patients with the baseline drug resistance tended to present with higher rates of virologic success if compared to the ones with drug susceptible viruses (90%, versus 30%, p = 0.02997, 2-sided Fisher's exact test), however this phenomenon was associated with higher adherence.

## Discussion

In this study we have evaluated the subtype D infections occurring among Caucasian individuals from Northwestern Poland. Differently to the cases of infection with this subtype observed across Europe [15], [17], [28], [29], the variant was found among native, non-immigrant and non-travelling Poles, and acquired locally. As is common in our country, HIV was diagnosed late, usually with symptoms of immunodeficiency and low baseline lymphocyte CD4 count [30]. Moreover, subtype D diagnosis in the advanced stages of the disease may be related to a faster clinical progression observed among patients infected with this subtype, lower lymphocyte CD4 count during follow-up and faster rates of lymphocyte CD4 decline, as previously described [31], [32], [33]. This may be the result of the increased degree of apoptosis in lymphocyte CD4 populations prior to the start of the antiretroviral treatment and a higher number of inhibitory programmed cell death receptors on the CD4 cells [34]. Mortality in the group was slightly higher if compared to the entire cohort (majority infected with the subtype B) from the region (3.44 versus 2.66 deaths per 100 person-years), which is in accordance to the previous reports [25], [35], [36], [37], [38]. Of note, all infections were associated with heterosexual transmissions with a percentage of infected women and mean age at diagnosis higher than among typical HIV positive population observed in Poland (75% versus 24.4%, respectively [26]). This may partially explain late diagnosis and as a consequence higher mortality and lower lymphocyte CD4 counts as heterosexual females, especially in peri- and postmenopausal period, are infrequently tested for HIV.

Observed infections with subtype D infections formed a network with probably one index case and subsequent local spread. This cluster of heterosexually acquired infections seemed independent from subtype B ones spreading among men who have sex with men (MSM),injection drug users (IDU) and heterosexual populations [27]. Dated phylogeny and phylogeographic analyses suggested that the tMRCA of the subtype D found in Poland is 1989.2 (95% HPD: 1994.46–1968.83) and is of Ugandan origin. The sequences cluster monophyletically within the sequences from Uganda, which confirmed a single transmission event in the past with the subsequent local spread. Local character of the transmissions was further confirmed by a lack of clustering with sequences from other European countries. Networks of transmission had been investigated both for B and non-B subtype infections using Bayesian MCMC inference methods, with identification of transmission clusters among MSM [2], [39] and heterosexual individuals [40]. Despite evidence of the slower dynamics among heterosexually acquired non-B subtypes, both small and large clusters with epidemiologically linked patients were found in the studies cited above. Similarly, in our study three small (two sequences) and one large (eleven) clusters were identified. Moreover, the Bayesian skyline plot indicates a stable population size. Dated phylogeny for the large cluster revealed that the majority of these transmissions occurred within the short period of time, probably in the early stage of infection.

Additionally, the high frequency of primary drug resistance mutations (DRMs) observed in this study was associated to the observed clustering within the network of transmission and presence of M41L and T215 revertant mutations. Similar results were reported in various countries for different subtypes, with most common transmission of NRTI DRMs [22], [41], [42], [43]. As expected, no integrase inhibitor (InI) transmitted resistance was found, which is consistent to the fact that all infections were acquired before introduction of InI in the clinical practice. We noted, that transmission of the drug resistance occurred both among individuals newly diagnosed, treatment-naive patients (cluster 4) and from a chronically infected, heavily treated patient (cluster 3). Both phenomena have been described previously [43], [44], [45], [46], [47], however this has been the first report on clustered drug resistance in Caucasians with subtype D. Non-R5 viruses were found infrequently in the group (in 13.6% when FPR 10% was used for the tropism assignment and 4.5% for 5.75 FPR), similarly to the result (14.8%) from a phenotypic assay performed in French patients [48]. Of note, our finding is in contrast to the studies demonstrating a high frequency of an ×4 tropism in Ugandan clades [49].

Immunologic and virologic antiretroviral therapy efficacy in the observed, stably treated group was poorer than expected for individuals on cART with no specific drug combination clearly superior in the group. Virologic failure was noted in 40% of cases, compared to the 24.7% in the European observational studies [50]. Association between poor adherence, treatment failure and development of secondary drug resistance was obvious, however the fact that the primary drug resistance did not affect the virologic treatment efficacy in the setting of good adherence must be acknowledged. It may be hypothesized that the poor adherence may be associated with the higher probability of cognitive impairment observed among individuals infected with this subtype [51]. Recently a pathogenetic mechanism of this phenomenon was proposed, as a certain features of the subtype D nef protein 3D structure was associated with long-term progressive dementia in HIV patients, suggesting that this cognitive deficit may be related to the altered folding or binding potential of this protein [52].

It has to be noted, that the limitations of this study can be related to the fact that the certain samples with subtype D might have remained undetected, as it was not possible to sequence all the samples from the patients followed-up in the centre. Neither index case, nor the certain source patient for the cluster 4 were found.

To sum up, local spread of HIV-1 subtype D infections described here represented an independent cluster, detected among heterosexually exposed individuals in parallel to the widely distributed subtype B infections. In phylogenetic inference analyses Ugandan origin of the virus was identified with probable single transmission event. Frequency of drug resistance in this group was high, especially within sub-clustered sequences, while the treatment efficacy was poor and independent of the primary drug resistance. Late diagnoses and association with heterosexual mode of transmission might fuel spread of these infections, especially in the light of evidence for the epidemiological clustering which suggests that new transmissions of HIV subtype D have been under diagnosed.

## Methods

### Population and ethics statement

For the study participants followed up at the Department of Infectious Diseases and Hepatology, Szczecin, Poland were recruited. The centre provides care for HIV infected patients, mostly from the Northwest part of Poland from the early 90-ties, and so far 960 patients have been observed. Clinical, epidemiological and laboratory data have been collected as a part of a routine patient care. The study has been approved by the local ethical committee of Pomeranian Medical University, Szczecin, Poland. All patients for whom sequence data (n = 190) were available have been analyzed. Treatment adherence was assessed based on the patient records (number of months of medications dispensed by the number of months of follow-up, expressed as a percentage) as well as clinical assessment based on the patient's statement regarding missed doses at home or treatment interruptions. Antiretroviral therapy was commenced according to the clinical standard at the time of introduction, stable therapy was defined as >30 consecutive days of uninterrupted treatment. Virologic success of the therapy was defined as stable serum viral load <400 copies/ml in the first available analysis following 6 months of treatment, and as a consequence no evidence of secondary drug resistance.

### Genotyping and subtyping

Plasma samples collected from patients between the years 1996 and 2011 were stored at −80 degrees C and analyzed retrospectively. HIV RNA extraction as well as reverse transcriptase and protease genotyping was performed using Viroseq 2.7 and 2.8 genotyping assays (Abbott Molecular, Abbott Park, IL) according to manufacturer's protocol. Initial subtyping was performed using REGA genotyping 2.0 tool (http://bioafrica.mrc.ac.za/rega-genotype/html/subtypinghiv.html) based on the partial *pol* sequence obtained by Viroseq methodology. Obtained protease/reverse transcriptase sequences were 1302 b.p. long; location from the start of HXB2 genome: positions 2253–3525. Subsequently, the subtype was confirmed in phylogenetic analysis with a selection of reference sequences listed in the HIV Sequence Compendium 2010 (Los Alamos National Laboratory, Los Alamos, U.S.A. http://www.hiv.lanl.gov). For this purpose bootstrapped (1000 replicates) neighbor-joining tree with Kimura 2 parameter model was used (MEGA version 4.0 [53]). To exclude recombinant sequences, bootscanning (200 base pair (b.p.) window, step 20 b.p., Kimura 2 parameter model) using Simplot software (S. Ray, John Hopkins University, Baltimore, USA) with a set of reference sequences was performed [54]. When necessary, samples from treatment-failing subtype D patients (genotyped for the purpose of drug resistance monitoring) were resequenced from the baseline (first available) sample. V3 sequences were obtained from both baseline and on-treatment samples while integrase was sequenced from the first samples only. HIV-1 integrase was amplified and sequenced using reagents and conditions specified by Laethem et al. [55], with the following amplification and sequencing primers: AGGAGCAGAAACTTWCTATGTAGATGG (outer forward), TTCTTCCTGCCATAGGARATGCCTAAG (outer reverse), TTCRGGATYAGAAGTAAAYATAGTAACAG (inner forward), TCCTGTATGCARACCCCAATATG (inner reverse and sequencing reverse), GCACAYAAAGGRATTGGAGGAAATGAAC (sequencing, forward), GGVATTCCCTACAATCCCCAAAG (sequencing, forward), GAATACTGCCATTTGTACTGCTG (sequencing, reverse). Obtained integrase sequences were 866 b.p. long, location from the start of HXB2 genome: positions 4230–5096. V3 loop sequencing was carried out following the protocol provided by the HIV Centre of Excellence (personal communication, prof. Richard Harrigan), with the following amplification and sequencing primers: GAGCCAATTCCCATACATTATTGT (outer forward), GCCCATAGTGCTTCCTGCTGCTCCCAAGAACC (outer reverse), TGTGCCCCAGCTGGTTTTGCGAT (inner forward), TATAATTCACTTCTCCAATTGTCC (inner reverse), AATGTCAGYACAGTACAATGTACAC (sequencing, forward), GAAAAATTCCCTTCCACAATTAAA (sequencing, reverse). Obtained integrase sequences were 107 b.p. long, location from the start of HXB2 genome: positions 7110–7217. Briefly, nested PCR was carried out following reverse transcription of extracted HIV-1 RNA as stated in the methodologies cited above. Amplicons were used for sequencing by standard techniques with BigDye technology using an ABI 3500 platform (Applied Biosystems, Foster City, CA, USA). V3 loop analyses were done in triplicate. Two overlapping sequencing reactions (forward and reverse) were performed for each sample. Reverse transcriptase and protease sequences were assembled with a software provided with a Viroseq 2.8 kit, while V3 and integrase sequences using the Recall online tool (http://pssm.cfenet.ubc.ca) [56]. Integrase and V3 sequences were obtained for all patients. Obtained V3 sequence data were interpreted using the geno2pheno tool (www.geno2pheno.org) with two FPR thresholds: 10% (as suggested by the European Guidelines on HIV Tropism testing) and 5.75% (from the Merit trial) [57], [58], [59].Drug resistance interpretation was performed using the Stanford DB database (hivdb.stanford.edu) [60] with transmitted HIV-1 drug resistance interpreted according to the surveillance recommendations [61], [62].

### Phylogenetic inference

To analyze the origin of our sequences, we have downloaded all HIV-1 group M subtype D *pol* sequences of at least 900 b.p. length, stored in the GenBank, using Los Alamos National Laboratory HIV sequence database (www.hiv.lanl.gov accessed on 16.09.2011). This dataset of 741 sequences was aligned with our partial *pol* sequences using Clustal ×2.0.10 (www.clustal.org). Bootstrapped (1000 replicates) maximum likelihood phylogeny using a GTR+γ model with empirical nucleotide frequencies was inferred using PAUP* v. 4.0 (http://paup.csit.fsu.edu/order.html). Separate analyses were performed for the dataset of 24 local sequences with maximum-likelihood methodology (bootstrapped with 1000 replicates) using PHYML v. 3.0 software with subtree pruning and regrafting (SPR) algorithm under GTR substitution model [63]. Moreover, to estimate the time to the most recent common ancestor (tMRCA) for local sequences we used a Bayesian Monte Carlo Marcov Chain (MCMC). Two replicates of 10 million generations were run in BEAST v. 1.5.3 [64], under the scenarios of the constant and exponential population size as well as Bayesian skyline plot using a GTR+γ+Γ and HKY models with uncorrelated lognormal relaxed molecular clock [1]. Log likelihoods for the models presented were as follows: −5790.27 for the relaxed a GTR+γ+Γ model with constant population size, −5790.27 for the relaxed a GTR+γ+Γ model with an exponential prior, −5833.38 for relaxed a GTR+2 codon positions with constant population size, −5837.73 for the HKY model. Models were compared using Chi-square distribution test, allowing to identify that GTR+2 codon position and HKY models were significantly less fit than the GTR+γ+Γ. As a GTR+γ+Γ models with constant population size and exponential priors were not statistically different, we have selected the scenario with the lowest marginal likelihood (constant population size). A consensus tree with posterior probabilities for branch support was obtained and annotated with TreeAnnotator v. 1.5.4. All trees were visualized in Figtree v.1.2.2.

### Statistics

Statistical comparisons were performed using the Fisher's exact t tests for nominal variables while continuous variables were analyzed using the U-Mann Whitney test using Statistica 8.0 PL software (Statasoft, Poland).

### Sequence data

HIV-1 subtype D sequences from this study sequences were submitted to the GenBank and may be accessed with the following IDs: GU906860, GU906864, GU906871, GU906872, GU906873, GU906874, JQ305750–JQ305792.
